# Proliferation-related changes in K^+^ content in human mesenchymal stem cells

**DOI:** 10.1038/s41598-018-36922-y

**Published:** 2019-01-23

**Authors:** Irina Marakhova, Alisa Domnina, Alla Shatrova, Aleksandra Borodkina, Elena Burova, Natalja Pugovkina, Victoria Zemelko, Nikolay Nikolsky

**Affiliations:** Department of Intracellular Signaling and Transport, Institute of Cytology, Academy of Sciences, St-Petersburg, 194064 Russian Federation

## Abstract

Intracellular monovalent ions have been shown to be important for cell proliferation, however, mechanisms through which ions regulate cell proliferation is not well understood. Ion transporters may be implicated in the intracellular signaling: Na^+^ and Cl^−^ participate in regulation of intracellular pH, transmembrane potential, Ca^2+^ homeostasis. Recently, it is has been suggested that K^+^ may be involved in “the pluripotency signaling network”. Our study has been focused on the relations between K^+^ transport and stem cell proliferation. We compared monovalent cation transport in human mesenchymal stem cells (hMSCs) at different passages and at low and high densities of culture as well as during stress-induced cell cycle arrest and revealed a decline in K^+^ content per cell protein which was associated with accumulation of G1 cells in population and accompanied cell proliferation slowing. It is suggested that cell K^+^ may be important for successful cell proliferation as the main intracellular ion that participates in regulation of cell volume during cell cycle progression. It is proposed that cell K^+^ content as related to cell protein is a physiological marker of stem cell proliferation and may be used as an informative test for assessing the functional status of stem cells *in vitro*.

## Introduction

Human mesenchymal stem cells (hMSCs) are adult stem cells derived from the mesenchymal tissues, such as bone marrow, adipose, dental pulp, amniotic fluid, endometrium^1–4^. Over the last few decades, they have gained much attention for their potential in clinical application. These cells are capable for self-renewal and differentiation into various lineages. hMSC may be maintained in culture for long periods without the loss of renewal capacity and ability to be differentiated under appropriate conditions. These adult stem cells are attractive not only as a potential source of cells in regenerative medicine, but also as a research tool in laboratory investigations. At present, an attention is focused on studies of signaling networks that regulate stem cell growth, differentiation, cell death. Little is known about the fundamental physiological properties of hMSCs, including the ion homeostasis and the role of ion transporters in maintaining cell survival and proliferation. Meanwhile, when investigating cellular response and signaling events in stem cells, it is important to deal with the functionally stable cells and to find the optimal conditions for cell maintaining *in vitro* and further manufacturing for clinical application.

Ion transporters and channels controlling cellular concentrations of monovalent ions have been shown to be important for cell growth and proliferation^5–10^. The expression levels of ion channels and ion pump have been found to differ in quiescent and transformed cells^11–17^. Inhibition of ion transporters with selective pharmacological drugs prevents the induction of cell proliferation in quiescent cells and induces cell cycle arrest in proliferating cell culture^18–22^. Unlike Ca^2+^, that is an important player in signaling network within the cell, the role of monovalent ions, such as K^+^, Na^+^, Cl^−^, in cell proliferation is not well understood. It is commonly suggested that changes in concentrations of Na^+^, Cl^−^ and H^+^ may play regulatory role in cell cycle progression. Changes in the cellular content of monovalent ions regulate intracellular pH (pH_i_) and transmembrane potential. It is proposed that cell Na^+^ concentration may affect the cell cycle progression by pH_i_ as well as altered Ca^2+^ signaling^23^. It has also been shown that Na^+^/H^+^ exchanger activity regulates G2/M progression by increasing pH_i_ which in turn regulates cyclin B1 expression and cdk2 activity^24–26^. Cellular Cl^−^ concentration may regulate cell cycle through cell membrane hyperpolarization and modulation of Ca^2+^ signaling during the G1/S transition^23,27^.

In previous studies, we have examined the changes in cell K^+^ and proliferative status of cultured cells. We have revealed significant changes in cell K^+^ content in long-term cultures of different cell lines: under optimal culture conditions, K^+^ content as calculated per cellular protein content was found to decrease in growing cultures of transformed cells of different origin^28–30^. The relationship between intracellular K^+^ content and cell proliferation was further examined in human blood lymphocytes which represent an adequate model for investigating the events underlying the transit of cell from quiescence to proliferation. We have found that cell K^+^ content per cell protein content was permanently increased during G0/G1/S transit: in mitogen-activated lymphocytes, the K^+^ content increase preceded the onset of DNA synthesis and was associated with the growth of small T cells into blasts^31–33^. The conclusion was made that cells that are preparing to proliferate are to raise their K^+^ content up to the higher level, and cell K^+^ content can be used as a physiological marker in determining the proliferative status of cell culture.

In this study, we focused on the ion homeostasis of human stem cells. We compared monovalent cation transport in hMSCs at different passages and at low and high density of cultures as well as during stress-induced cell cycle arrest and revealed proliferation-related changes in K^+^ content per cell protein and K^+^ influxes via Na^+^, K^+^-ATPase pump. Our present study highlights the importance of K^+^ as the main intracellular ion for successful proliferation and suggests that the cell K^+^ content as related to cell protein is a functional characteristic for stem cell proliferation. The mechanism which is potentially involved in the proliferation-associated changes in cell K^+^ content is suggested.

## Results

### Intracellular K^+^ and Na^+^ content during the growth of hMSC culture

**Figure 1:**
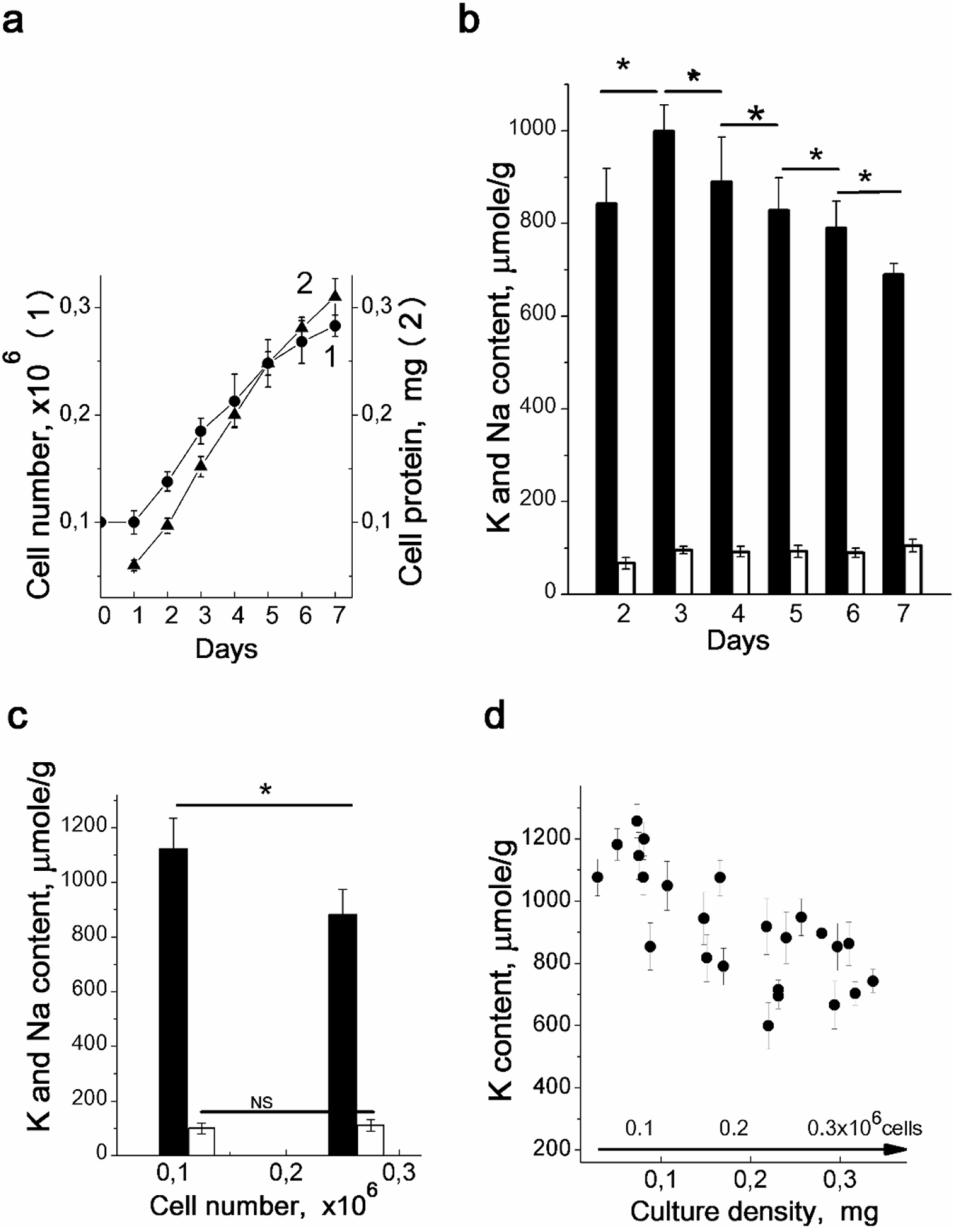
Density-dependent changes in intracellular K^+^ and Na^+^ content during the growth of hMSCs culture. **(a)** Growth curve (1) and cell protein content (2) in cultivated hMSCs. A representative data of seven independent experiments are presented. **(b)** Changes in intracellular K^+^ and Na^+^ content per cell protein during the growth of hMSCs culture. The same experiment as in (**a**). **(c)** Increased culture density impact on K^+^ content in hMSCs. hMSCs (passage #2) were seeded simultaneously at two densities (5 × 10^4^ and 15 × 10^4^ cells per 35 mm dish), and at the third day, the intracellular cations were estimated. Data are representative of three independent experiments. **(d)** Cell K^+^ content decreased with increasing cell density in culture. Summary data from thirteen independent experiments are presented. The experimental conditions are similar to those in (**a**) and (**b**). The culture density is presented as cell protein per 35 mm dish with hMSCs culture. Bar graphs indicate the mean ± SD, n = 3–4. *p < 0.05 by Tukey *t*-test for each pair of columns. NS, not significant.

To characterize the ion homeostasis of cultivated hMSCs, K^+^ and Na^+^ contents were evaluated in cells during culture growth from low to high density. After initial delay during the first day after seeding, the hMSCs were exponentially growing during the next 6 days (Fig. 1a). In growing hMSCs culture, the amount of cell protein (used as an additional indicator of cell number increase in the same culture) was also augmented (Fig. 1a). It was noticed that in dense cultures with declined cell multiplication rate the cell protein mass continued increasing. As a result, in confluent culture of hMSCs the protein content per single cell was higher than in sparse and sub-confluent culture.

In adherent cell culture, there are considerable difficulties in calculating the intracellular ion concentrations because of uncertainties in measuring cell volume and water content: cells have different sizes and it is impossible to compare cells of different lines. To evaluate cellular content of ions in growing hMSCs culture, we normalized cell ion content per cell protein mass in the same culture. In cell biology studies, such method for calculating intracellular ion concentration is widely used. We revealed that cell K^+^ content per cell protein was changed during hMSCs cultivation. After increasing within the first 2 days after plating, later K^+^ content starts to decrease. As shown in Fig. 1b, K^+^ content diminished from 999 ± 57 µmole/g (n = 4) on the 3d day to 690 ± 24 µmole/g (n = 3) on the 7th day of culture maintenance. The decrease in cell K^+^ content was not associated with a medium depletion since a change to fresh medium on the 2nd and the 4th days after plating had no effect on the K^+^ content decrease. Thus, during a single passage, hMSCs growing in high densities show a significant decrease in K^+^ content per cell protein. In growing culture of hMSCs, intracellular Na^+^ content did not change as much as K^+^ and the changes observed were not regular: Na^+^ content per cell protein was 95 ± 8 µmole/g (n = 4) at the 3d day, 93 ± 13 (n = 3) at the 5th day and 105 ± 14 µmole/g (n = 3) at the 7th day of cell cultivation (Fig. 1b). At all stages of culture growth, hMSCs are characterized by high intracellular K^+^/Na^+^ ratio typical for most animal cells.

To find out whether the decrease in K^+^ content in proliferating hMSCs was caused by prolonged cultivation, we prepared sparse and confluent cultures simultaneously: cells were seeded at the same day, but with different densities, and cell ion contents were estimated at the same day after plating. As shown in Fig. 1c, cell K^+^ content was lower in high density culture (250 × 10^3^ cells per dish) than in low density culture (100 × 10^3^ per dish). We concluded that during the maintenance of MSCs under optimal culture conditions, changes in cell K^+^ content were not due to the time in culture, but in growing culture a decrease in intracellular K^+^ content might de due to the higher cell density in culture (Fig. 1d).

### Rb^+^ influx decreases during the growth of hMSC culture

**Figure 2:**
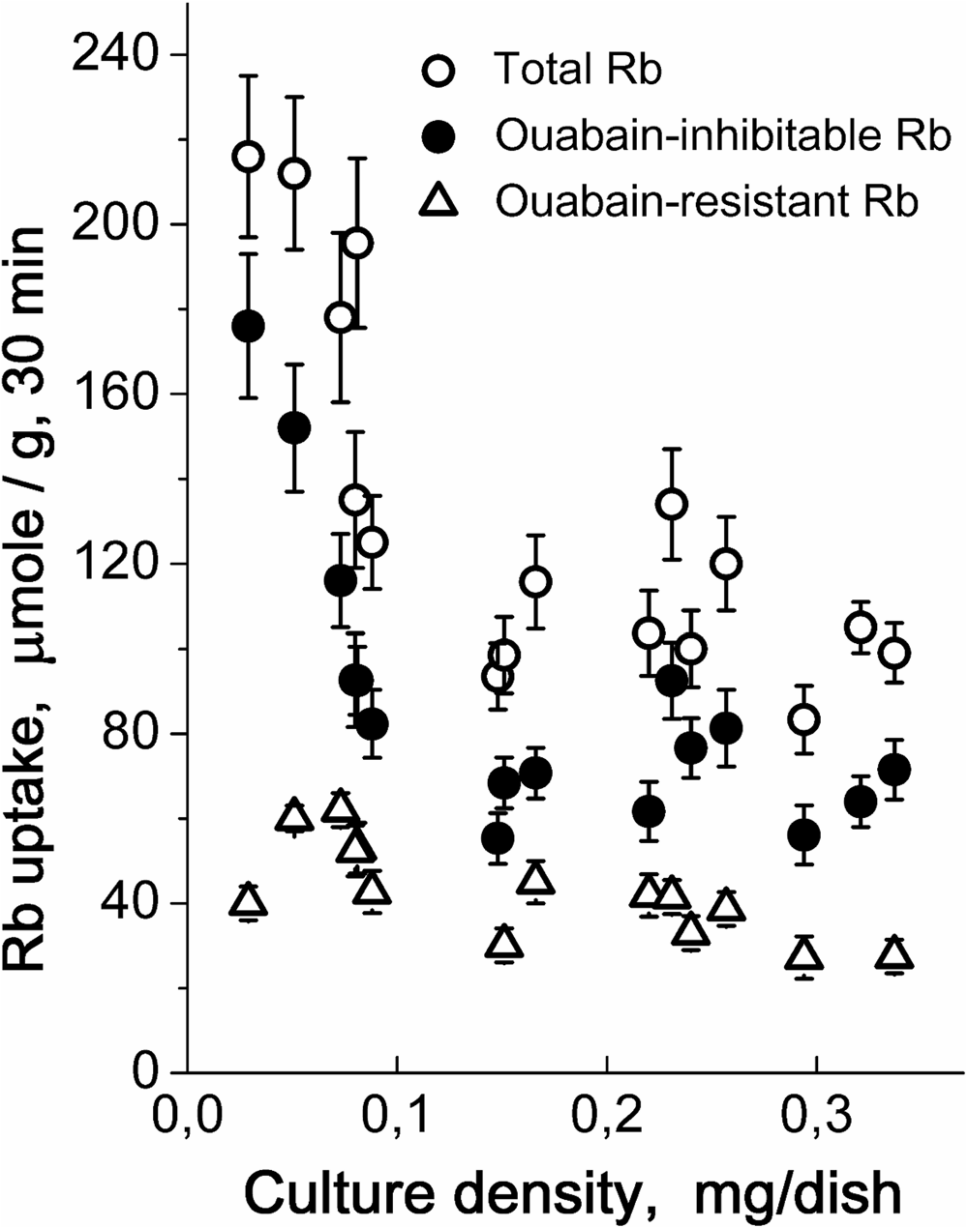
Rb^+^ influx in hMSCs is dependent on the cell density in culture. Rb^+^ influxes were defined in one experiment for three cultures of the same passage. *Open circles*: total Rb^+^ uptake; *filled circles*: ouabain-inhibitable Rb^+^ influx; *triangles*: ouabain-resistant leakage. The experimental conditions are similar to those presented in Fig. 1b. Every point represents the mean ± SD, n = 3–5. Summary data of thirteen independent experiments.

A short-term Rb^+^ uptake was used to evaluate the changes in K^+^ flux across plasma membrane. We revealed that during the culture growth, Rb^+^ influx was not constant. As with cell K^+^, Rb^+^ influx decreased during the growth of cell culture to confluency (Fig. 2). In hMSCs, the ouabain-inhibitable Rb^+^ influx, mediated by the Na^+^, K^+^-ATPase pump, comprised more than a half of the total Rb^+^ uptake and Rb^+^ uptake changes during culture growth were mainly due to a decrease in ouabain-inhibitable influx. Under the optimal conditions, within the first 3 days the density of cell culture was usually doubled and the ouabain-inhibitable Rb^+^ uptake decreased from 106 ± 16 (n = 6) to 72 ± 5 (n = 7) µmole/g, 30 min. These data may indicate that in hMSCs, the functional expression of the Na^+^, K^+^-ATPase pump was down-regulated with increasing cell density in culture. At the same time, the ouabain-resistant Rb^+^ influx (i.e. K^+^ leakage) remained unchanged (Fig. 2).

### Age-dependent changes in K^+^ and Na^+^ content in MSC culture

**Figure 3:**
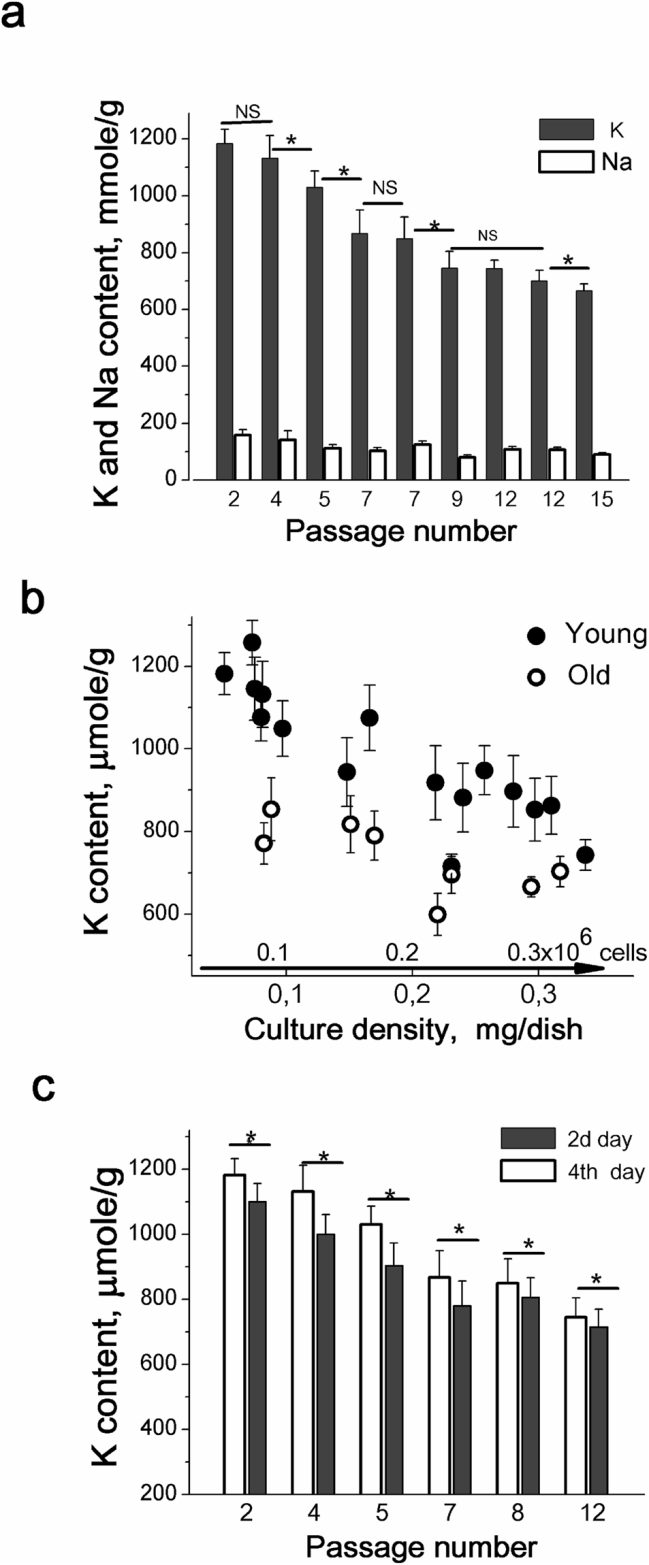
Intracellular K^+^ content is dependent on the passage number of hMSCs. **(a)** Cell K^+^ content per cell protein is decreased in the late-passaged hMSCs. *Filled bars*: cell K^+^ content, *open bars*: cell Na^+^ content. The data are presented as mean ± SD, n = 3. p < 0.05 by Tukey *t*-test for each pair of columns. NS, not significant. **(b)** Density-associated decrease in cell K^+^ content in early-passage (*filled circles*) and in late-passage (*open circles*) hMSCs. Summary data of eleven independent experiments are presented. Every point corresponds to cell K^+^ content determined in one experiment for cultures of the same passage. The data are presented as mean ± SD of three individual cultures of the same passage. **(c)** Density-associated decrease in cell K^+^ content is more significant in early-passage hMSCs as compared to late-passage hMSCs. *Open bars*: K^+^ contents in sparse cultures (at the second day); *filled bars:* K^+^ contents in confluent cultures (at the fourth day). All data are presented as mean ± SD, n = 3, *p < 0.05 by Tukey *t*-test for each pair of columns.

We analyzed cells at different passages and revealed that cell K^+^ content was dependent on cell age. It was higher in “young”, early-passage than in “old”, late-passage cultures. Figure 3 represents the data on K^+^ content in hMSCs of different passages. In cells at the 2nd–5th passages, K^+^ content per cell protein was higher that of in cells at the 12th–15th passages (Fig. 3a). Moreover, a degree of K^+^ content decrease during the growth of cell culture to confluence was also dependent on the age of culture (Fig. 3c). In the early-passage hMSCs (up to 7 passages) the decrease in K^+^ content in high-density culture was larger (12–15%) as compared to the K^+^ content decrease in the late-passage hMSCs (4–7%). In contrast to K^+^, changes in Na^+^ content in cells of different passages were not regular and we did not find any significant differences in cell Na^+^ content between “young” and “old” cells (Fig. 3a). Altogether, the above findings indicate that intracellular K^+^ content during the maintenance of hMSCs in culture is dependent on both the cell density and the age of the cell culture (Fig. 3b).

### Changes in cell K^+^ content and proliferative status of hMSCs cultures

**Figure 4:**
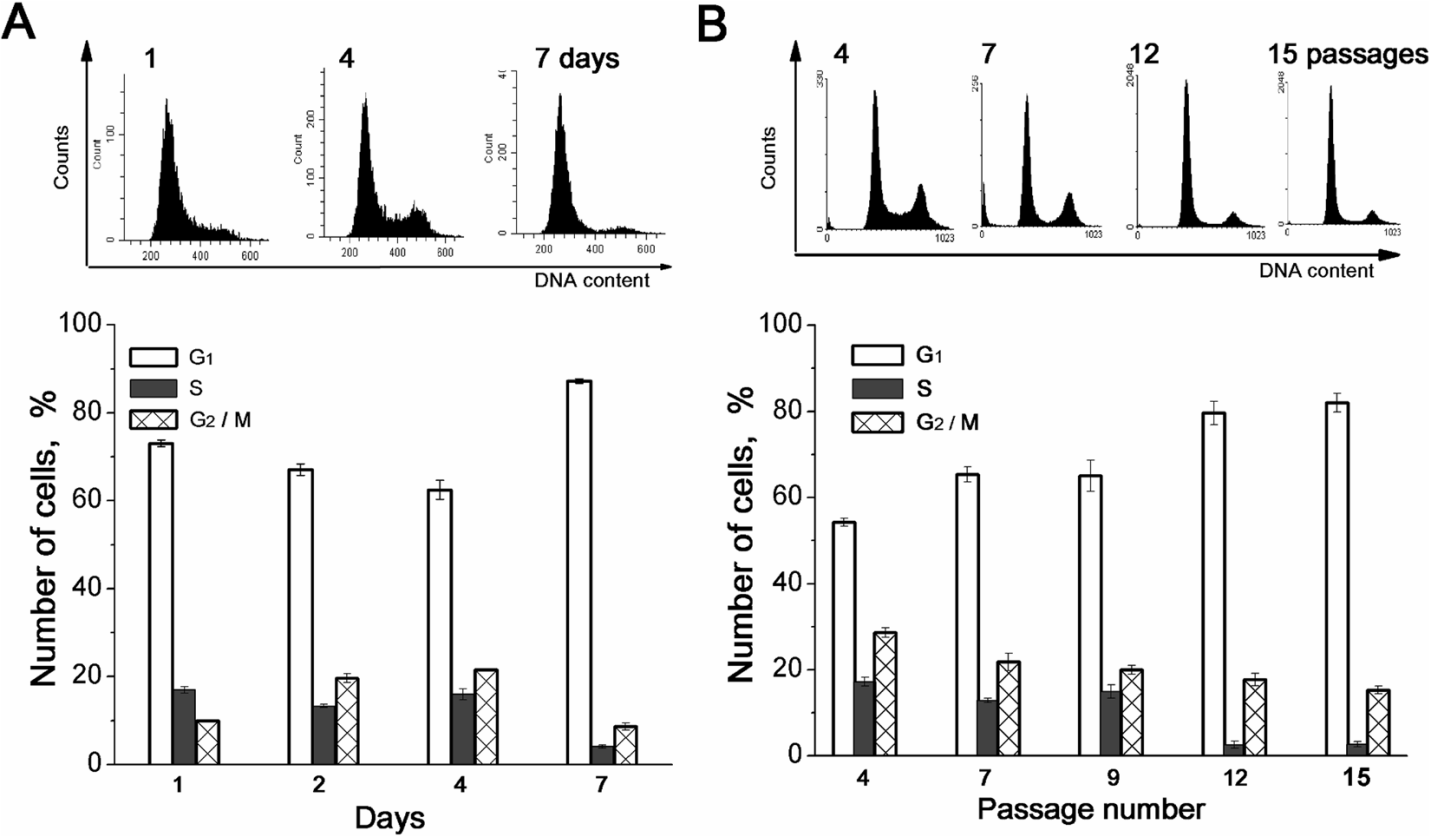
Cell proliferation rate is decreased both in the high density and in the late passage hMSCs cultures. **(a)** Cell cycle profile of hMSCs growing during seven days in culture. FASC assay (*upper panel*) and the percentage of cells in G1, S and (G2 + M) phases (*low panel*). A representative data of one experiment from five performed on the same scheme. **(b)** Cell cycle profile of hMSCs is dependent of the passage number. FACS assays (*upper panel*) of cultures at the third day after plating and the percentage of cells in G1, S and (G2 + M) phases (*low panel*). A representative data of nine experiments performed on the same scheme. All data are presented as mean ± SD of three individual cultures.

In previous studies we suggested that in growing cultures of different cell lines, the changes in intracellular monovalent cations were due to a decrease in the proliferation rate in confluent cell cultures^29,30^. Therefore, we next analyzed cell cycle profiles and found that at the 7th day of cell cultivation, there was an accumulation of cells in G1 phase and a decrease in S and G2/M phases (Fig. 4a). Thus, the cell cycle profile in MSCs cultures of high density showed that the cell population was slowing down their proliferation. When comparing the changes in cell ion contents and cell cycle profiles in the course of hMSC cultivation, we found that K^+^ content per cell protein decreased simultaneously with the increase in G1 cell population. In contrast, growth-related decrease in proliferation status of MSCs was not accompanied with changes in cell Na^+^ content.

The correlation between а decrease in cell K^+^ content and accumulation of cells in G1 phase was seen in hMSC cultures of different passages. The analysis of cell cycle profiles showed that in early-passage sub-confluent cell culture (at the 2–4th passages) up to 45% of cell population was in (S + G2 + M) phases, whereas phase profile of late-passage cell population (above 10–12 passages), was characterized with accumulation of cells in G1 phase (81,9%) (Fig. 4b). Together, the above data suggest that in hMSCs, density- and age-dependent decrease in K^+^ content is related the delay in cell cycle progression and to the inhibition of cell proliferation.

To explore relationships between K^+^ content per cell protein and proliferation of hMSCs we tried to change intracellular K^+^ content by manipulating the permeability of cell membrane for K^+^ and tested the effect of pharmacologically inhibiting K^+^ influx on intracellular K^+^ content and proliferation rate. We asked whether K^+^ channel blockers are able to decrease cell K^+^ content per cell protein and to slow down cell proliferation.

**Figure 5:**
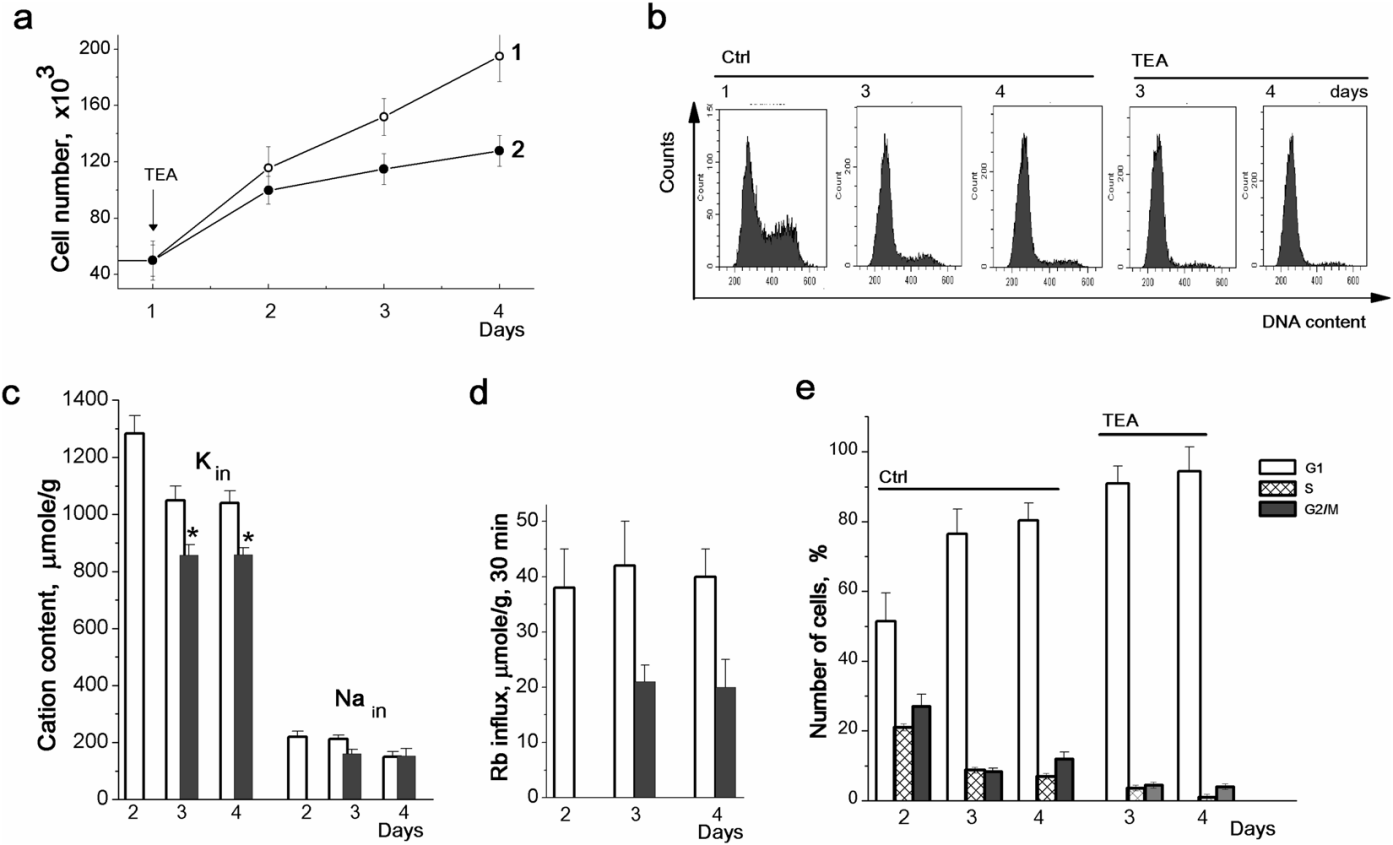
TEA effects on growth, cation content and proliferation of hMSCs. A representative data of two experiments performed on the same scheme. **(a)** Growth curves of control hMSCs (1, *open circles*) and hMSCs treated with 10 mM TEA (2, *filled circles*). **(b)** Cell cycle profiles of hMSCs growing four days in complete culture medium or in the presence of 10 mM TEA. **(c)** and **(d)** Effect of 10 mM TEA on cell K^+^ and Na^+^ content (**c**) and ouabain-resistent Rb^+^ influx (**d**). *Light* bars: untreated cells; *gray* bars: TEA-treated cells. **(e)** TEA increases the number of cells at G1 phase. The data are presented as mean ± SD (n = 3), *p < 0.05 by Tukey t-test, versus untreated cells. Ctrl: untreated cells.

In experiments, we used tetraethylamminium chloride (TEA) as a blocker of K^+^ channels in cell membrane. TEA was added to culture medium at the 2nd day after cell plating and K^+^ and Na^+^ contents as well as Rb^+^ influxes were determined during culture growth. We observed that TEA (10 mM) inhibited the growth of MSCs culture one day after addition to culture medium (Fig. 5a). Four days after TEA addition there were 115 ± 11 × 10^3^ cells/plate (n = 3) compared to 195 ± 18 × 10^3^ cells/plate (n = 3) in control. To investigate if TEA can affect the survival of hMSCs, the percentage of viable cells was studied using the propidium iodide exclusion assay. We found that on the 4th day in TEA-treated cultures, the percentage of viable cells in population was 91 ± 7 (n = 3) instead of 96 ± 5 (n = 2) in untreated cells.

As seen in Fig. 5c, in cultures with TEA intracellular K^+^ was lower that of in cultures without TEA. Ouabain-resistent Rb^+^ influxes in TEA-treated cells were half less than in control cells (Fig. 5d). To the last day of cell cultivation with TEA^+^ internal Na^+^ content was comparable with that in control (Fig. 5c). Thus, a significant decrease in passive K^+^ leakage across cell membrane as well as in cell K^+^ content per cell protein was observed in hMSCs in the presence of TEA. Notably, in TEA-treated cells, the cellular K^+^/Na^+^ ratio (as an indicator of normal and high ion heterogeneity) remained high (5,2–5,6).

As found by flow cytometry measurements, treatment with 10 mM TEA increased the percentage of cells in G1 phase and decreased that in S and G2/M phases to 94 ± 7 (n = 3) and 5,0 ± 0,8 (n = 3) respectively (Fig. 5b,e). Thus, reduction of K^+^ leakage leads to decrease in cell K^+^ content per cell protein and to cell cycle delay at G1 phase.

### K^+^ transport and H_2_O_2_-induced cell cycle arrest

To explore further the relationship between intracellular K^+^ and cell proliferation we examined ion changes during the stress-induced proliferation arrest and the development of premature senescence. Senescence was shown to be an irreversible cell cycle arrest of metabolically active cells^34–38^. We asked whether changes in K^+^ transport accompany also the growth arrest during the premature senescence and investigated the ion changes in hMSCs stimulated by sublethal doses of H_2_O_2_. H_2_O_2_ is an intermediate product of cellular metabolism. Therefore, it is widely used in studies of oxidative stress in cell culture models.

**Figure 6:**
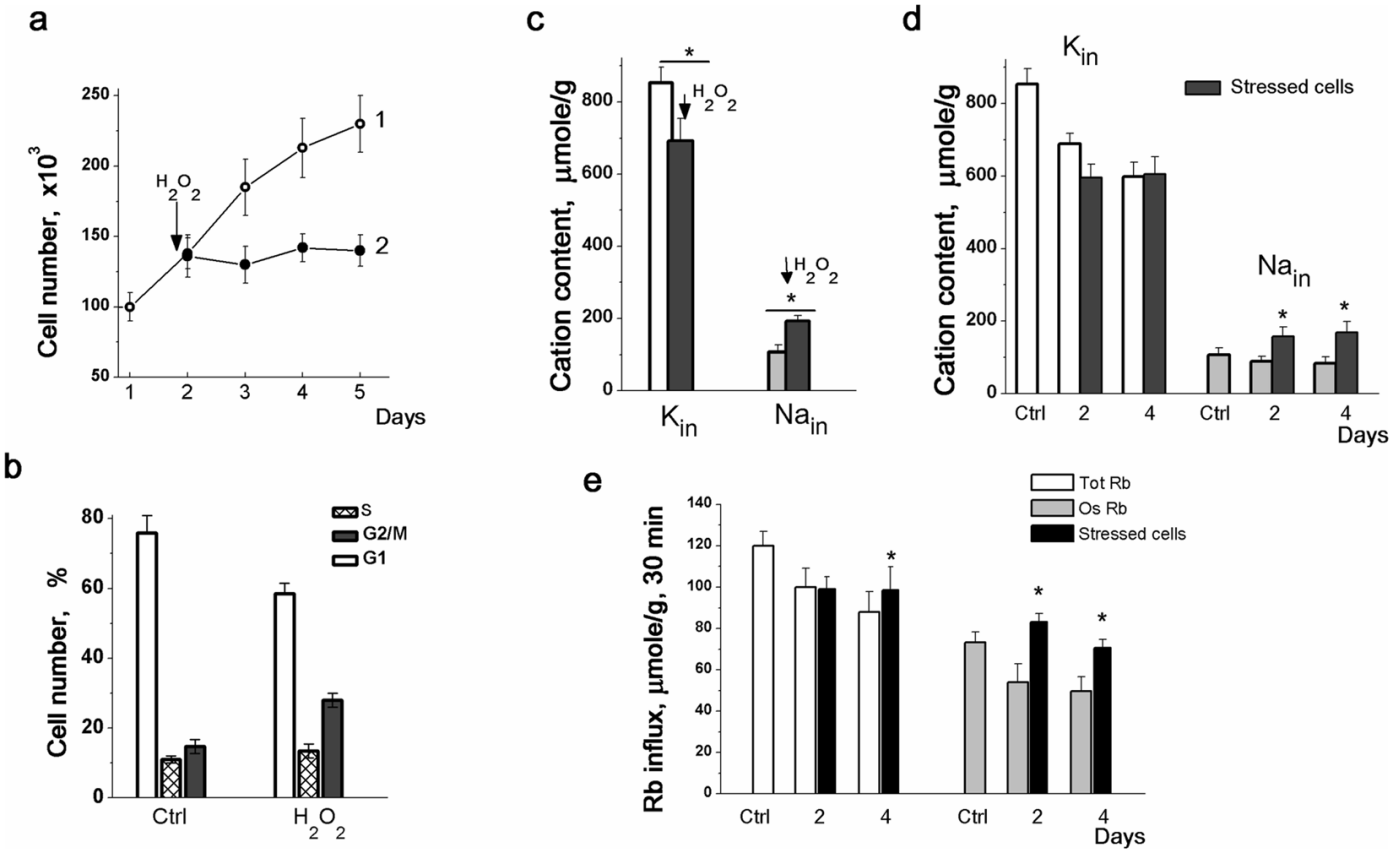
Changes in K^+^ and Na^+^ content and Rb^+^ influxes in hMSCs and H_2_O_2_-induced cell cycle arrest. **(a)** Growth curves of control (1, *open circles*) and H_2_O_2_-treated (2, *filled circles*) hMSCs. A representative data of four experiments performed on the same scheme. **(b)** H_2_O_2_-treated hMSCs exhibited cell cycle arrest in G2/M phases. Cell cycle profile of hMSCs growing four days after H_2_O_2_ pulse under normal culture conditions is presented. Ctrl: untreated cells. **(c)** Changes in K^+^ and Na^+^ content in H_2_O_2_-treated hMSCs. An arrow indicates the H_2_O_2_ “pulse” (200 μM H_2_O_2_ for 0.5 h). *Light* (K_in_) and *light grey* (Na_in_) *bars*: untreated cells; *black bars*: H_2_O_2_-treated cells. Data are presented as mean ± SD, n = 3, *p < 0.05 by Tukey *t*-test in each bar graph. **(d)** H_2_O_2_-induced cell cycle arrest is accompanied by persistently elevated Na^+^ content and did not affect K^+^ content in hMSCs. *Light* (K_in_) and *light gray* (Na_in_) bars: untreated cells; *black bars* – after H_2_O_2_ “pulse” cells were cultivated under normal conditions for the indicated days. Ctrl: untreated cells. **(e)** Changes in Rb^+^ influxes in H_2_O_2_-treated hMSCs. *Light bars*: total Rb^+^ uptake; *light gray bars*: ouabain-sensitive Rb^+^ influx, *black bars*: H_2_O_2_-treated cells. The data are presented as mean ± SD (n = 3), *p < 0.05 by Tukey *t*-test, versus unstressed cells. Ctrl: untreated cells.

Earlier, it was shown that hMSCs treated with sublethal doses of H_2_O_2_ enter the premature senescence accompanied by the irreversible cell cycle arrest, cell hypertrophy and enhanced SA-β-Gal staining^39^. We confirmed that the growth of hMSCs, shortly subjected to 200 μM H_2_O_2_ and then returned to the normal culture conditions, was stopped. As presented in Fig. 6a, 3 days after the H_2_O_2_ pulse (1 h), the cell number remained unaltered whereas the population of control cells was increased. In our experiments, H_2_O_2_-treated cells were arrested, at least, for 5 days. The results demonstrate cellular hypertrophy in response to H_2_O_2_, namely, growth arrest was accompanied by protein content elevation so that the amount of protein per single cell was increased (Fig. 6b). After short-term treatment with H_2_O_2_, hMSCs retain high viability. As evaluated by FACS analysis, in 96 hours after H_2_O_2_ treatment, the percentage of viable cells in population was 86 ± 7 (n = 3) instead of 96 ± 5 (n = 3) in untreated cells. Cell cycle analysis by flow cytometry revealed that H_2_O_2_-treated cultures were arrested mostly in G2/M transition (Fig. 6c). Based on these observations we concluded that in our experimental conditions, hMSCs, which received short-term sublethal H_2_O_2_ pulse may be used as a model for studies of ion homeostasis during stress-induced cell cycle arrest.

First, we examined cation changes in hMSCs in response to the sublethal concentration of H_2_O_2_ and revealed a significant decrease in K^+^ content and increase in Na^+^ content after 1 h treatment with 200 μM H_2_O_2_ (Fig. 6d). As a result, intracellular K^+^/Na^+^ ratio decreased from 7–8 to 3–3.5 thus indicating the disturbances in cellular ion homeostasis. After replacing the medium to H_2_O_2_-free serum-containing DMEM, cell K^+^ content gradually increased, cell Na^+^ content decreased and by the next day after H_2_O_2_ pulse, hMSCs restored their ion heterogeneity and high intracellular K^+^/Na^+^ ratio.

When testing the delayed ion changes in H_2_O_2_-treated cells we revealed that 4 days after short H_2_O_2_ treatment, in arrested culture, intracellular K^+^ content per cell protein remained high and did not differ from that of in control cells whereas Na^+^ content was doubled (Fig. 6e). The H_2_O_2_-induced cell cycle arrest in hMSCs was also accompanied by an increase in ouabain-inhibitable Rb^+^ uptake. This implies that under sublethal oxidative stress, the pump-mediated K^+^ influx was enhanced, presumably due to the higher intracellular Na^+^ concentration. Such ionic changes (elevated Na^+^ content, increased ion pumping and decreased cell K^+^/Na^+^ ratio) can indicate the physiological damage of stressed cells.

## Discussion

In this study, we report changes in intracellular K^+^ and Na^+^ contents as well as in transmembrane K^+^ influxes during the growth of stem cell culture. First, we observed that within a single passage, hMSCs grown to high densities showed a significant decrease in K^+^ content per cell protein mass. At higher densities of cell monolayer, the ouabain-sensitive K^+^ influx was decreased thus indicating a decline in Na^+^, K^+^-ATPase pump-mediated transport. Next, we revealed the age-dependent changes in ion transport in hMSCs. In early-passage hMSCs, K^+^ content per cell protein and ouabain-inhibitable K^+^ transport were higher as compared to late-passage hMSCs. We analyzed the cell cycle profiles of hMSCs under different growth conditions and concluded that in high-density cultures as well as in late-passage hMSCs, the decline of K^+^ content per cell protein was associated with accumulation of G1 cells in population and accompanied cell proliferation slowing.

We examined also ion homeostasis in stress-induced hMSCs and revealed that H_2_O_2_-induced cell cycle arrest and senescence development was accompanied by elevated intracellular Na^+^ content and decreased cell K^+^/Na^+^ ratio. No specific changes in intracellular K^+^ content were found in stress-arrested hMSCs as compared to that in growing culture. Worth mentioning, senescent cells displayed about 2 times higher Ca^2+^ concentration as compared to control cells^35,40,41^. There are also findings implicating NHE1 (Na^+^/H^+^ exchanger) activation as a signaling event activated by stress conditions and modulating cell proliferation and death^42^. Altogether, the data available suggest that stress-induced senescent cells are characterized by changes in ion homeostasis that indicate physiological damage of cells and are prerequisite to cell death.

At present, there is little information on ion homeostasis and the role of ion transporters in stem cell proliferation and differentiation. Some studies have shown the existence of various ion channels in hMSCs^43,44^. Several K^+^ and Na^+^ channels at the mRNA and functional levels were revealed in hMSCs from umbilical cord vein channels^45^. Cardiomyocytes derived from mouse embryonic stem cells have been demonstrated to exhibit a time-dependent expression of ion channels^46^. Expression and distribution of Na^+^, K^+^-ATPase subunits are changed during differentiation of pluripotentent embryonic stem cells^47,48^. It has been revealed that stem cells could increase the activities of the sarcolemma Na^+^, K^+^-ATPase and the sarcoplasmic reticulum membrane Ca^2+^-ATPase in heart failure, a possible mechanism to improve heart function^49,50^.

Our study was focused on relations between K^+^ transport and stem cell proliferation. Recently, using reflection X-ray fluorescence spectrometry and mass spectrometry, the extensive research of the inorganic components in human stem cells in distinct states of cellular pluripotency was performed^51^. This study revealed that intracellular K^+^ content differs considerably between non-pluripotent and pluripotent cells (hPSCs), and the perturbations of K^+^ homeostasis in the presence of pharmacological drugs affect the intracellular signaling in hPSCs and the cell reprogramming for induced hPSCs production.

Many evidence indicate that inhibition of Na^+^, K^+^-ATP-ase pump with ouabain or in low K^+^ medium and the subsequent decrease in intracellular K^+^ content reduced the growth rate in cultures of permanent cell lines^19,52,53^. It was demonstrated that the decrease in cell K^+^ content below some threshold level (about 500 µmole/g proteins) stopped proliferation^54,55^. The asymmetric distribution of Na^+^ and K^+^ is a universal characteristic of living cell and high cellular K^+^/Na^+^ ratio is necessary for successful proliferation.

In our study of MSCs cultures, we discovered growth-dependent cell K^+^ decline within a range from 1,100 to 600 µmole/g protein that was associated with accumulation of G1 cells in population and accompanied proliferation delay. To elucidate the relations between growth-dependent cell K^+^ changes and proliferation we asked whether higher cell K^+^ per cell protein might be essential for maintaining cell proliferation in the context of volume regulation of cycling cell.

During cell cycle, an increase of cell volume is required: before division, the cycling cell is growing and its volume is significantly increased^56^. In experiments with modulations of osmotic cell balance it was found that cell swelling promoted and cell shrinkage inhibited proliferation^57,58^. Cells are known to contain impermeable anionic macromolecules (such as proteins, nucleic acids, etc.) that set up an unstable osmotic condition and could lead to cell lysis. Animal cells cope with this problem and prevent the water flows into the cell, induced by intracellular impermeable molecules, by pumping Na^+^ out and K^+^ in; simultaneously, a coupled transport of osmolytes (including highly permeable monovalent ions, such as K^+^, Na^+^, Cl) is another factor that is responsible for cell volume regulation (“pump-leak model”)^59–64^.

During cell cycle, the amount of osmotically active substances significantly increases that induces an increase in cell water content and needs the participation of ion movements through cell membrane. Ion channels (in particular, K^+^ channels) play an active role in cell cycle progression and participate in adjustment of cell volume^65–69^. An inhibition of K+ channel can retard the cell cycle^22,45,70^. In our experiments with hMSCs, K^+^ channel blocker TEA decreased inward Rb^+^(K^+^) leakage and TEA-induced cell cycle delay was associated with a decrease in cell K^+^ content per cell protein. Here, we wonder, what could be the functional significance of changes in K^+^ content/protein content ratio for growing cell cultures. Really, this parameter may have a specific meaning for living cells in which the protein mass increases.

According to the theory of cell ion and water balance in animal cells, cell K^+^ content per cell protein correlates always with cell water content per cell protein as soon as K^+^ is the major cellular osmolyte^61,63,71–74^. To date, few studies are available on changes in cell volume and K^+^ content in cycling cell. As shown in synchronized Ehrlich ascites cells, K^+^ concentration (i.e. K^+^ content per water content) does not change during cell cycle progression^23^. However, it is well known that the cell buoyant density (i.e. cell dry mass per cell water content) is much higher in proliferating than in quiescent cells^30,64^. In the present study, we found that growth-associated cell K^+^ decline represents K^+^ content change related to cell protein. Similar changes in K^+^ transport were earlier revealed in cultures of permanent cell lines^29,30^. It was also shown that in mitogen-activated lymphocytes, G0/G1 → S transit and the growth of small T cells into blasts is accompanied by a gradual increase in the ratio of cell K^+^ content per cell protein^31–33^. Altogether, these observations may indicate that in cycling cells the water content per cell protein should be higher than that in quiescent or differentiated cells. It is interesting to note here that there is evidence that the relationship between cell volume, cell water and proliferation rate can reflect the effects of macromolecular crowding on enzyme activity and cell metabolism^58,75^.

In summary, the mechanisms by which monovalent ions regulate cell proliferation seem to be different. Ion channels and ion transporters may be implicated in signaling in cells stimulated by growth-promoting factors. Changes in free, monovalent ion concentration regulate cellular pH_i_, transmembrane potential, Ca^2+^ homeostasis and the activity of some proteins with important function in cell cycle progression. Our study is the first to elucidate the relationship between cell K^+^ content as calculated per cell protein and successful proliferation of stem cells. We revealed the changes in intracellular K^+^ content per cell protein that are closely associated with growth and proliferative status of hMSCs. Based on these results and previous studies of monovalent ion transport in permanent cell lines and human T cells, we suggest (1) cell K^+^ may be important for cell proliferation as the main intracellular ion that is involved in cell volume regulation during cell cycle progression, and (2) in cycling cells, the water content per cell protein should be higher than that in quiescent or differentiated cells. It is proposed that cell K^+^ content as related to cell protein is a physiological marker of stem cell proliferation and may be used as an informative test for assessing the functional status of stem cells *in vitro*.

## Methods

### Cells and cell treatment

Experiments have been performed on hMSCs (lines 2304 and 2804) obtained from Department of intracellular signaling and transport of Institute of cytology RAS (Russia)^76^. No donor consent was needed.

The cells are multipotent, capable for self-renewal, express CD13, CD29, CD44, CD73, CD90, CD105 and are negative for the hematopoietic markers CD19, CD34, CD45, CD117, CD130, and HLADR (class II)^29^. Multipotency of isolated hMSCs is confirmed by their ability to differentiate into other mesodermal cell types, such as osteocytes and adipocytes. Besides, the isolated hMESCs partially (over 50%) express the pluripotency marker SSEA-4. These cells are characterized by high rate of cell proliferation (doubling time 22–23 h) and high cloning efficiency (about 60%). Cells were maintained in Dulbecco’s Modified Eagle Medium (DMEM)/F12 (Gibco) supplemented with 10% fetal calf serum (HyClone), 1% penicillin-streptomycin (Gibco BRL, MD, USA), and 1% glutamax (Gibco BRL, MD, USA) and subcultured at 1:3–1:4 ratio twice a week. For experiments, MSCs were seeded into 35-mm Petri dishes (10 × 10^4^ cells per dish). Cells were passed every 5–7 days. The culture medium was changed every day until the collection of cells for analyses at each time point. Cells at the 2th–15th passages were used in the experiments.

H_2_O_2_ treatment was performed on the subconfluent cells. H_2_O_2_ stock solution in serum-free medium was prepared from 30% H_2_O_2_ (Sigma, USA) just before adding. Cells were treated with 200 μM H_2_O_2_ for 1 h, then washed twice with serum-free medium to remove H_2_O_2_, and re-cultured in fresh complete medium for various durations as specified in individual experiments. The short-term treatment with 200 μM H_2_O_2_ was sublethal and was shown to induce senescence in hMSCs^39^. In separate set of experiments, tetyraethylammonium chloride (TEA, Sigma) used as K^+^channel blocker, was added to cell culture at the 2nd day after plating at final concentration of 10 mM.

### Analysis of cell K^+^ and Na^+^ content and K^+^ influx

Measurements of ions were performed essentially as described previously^31^. To estimate K^+^ influx Rb^+^ was used as the physiological analog of K^+^. RbCl (final concentration 5 mM) was introduced into the culture medium for 20 min. To evaluate the Na^+^, K^+^-ATPase K^+^ influx, prior to RbCl, ouabain (10^–4^ M) was added to medium. Then cells were rapidly washed 5 times with ice-cold isotonic MgCl_2_ and cations were extracted with 1 ml of 1% trichloroacetic acid (TCA). TCA extracts were analyzed for Rb^+^, K^+^ and Na^+^ by emission flame photometry. TCA precipitates were dissolved in 0.1 N NaOH and analyzed for protein by Lowry procedure. Ouabain-sensitive Rb^+^ uptake was calculated as the differences between the mean values measured in samples incubated with and without ouabain. The intracellular ion content was expressed as amount of ions per amount of protein in each sample analyzed.

### FACS analysis of cell cycle progression and cell viability

Adherent cells were rinsed twice with PBS and harvested by trypsinization. Detached cells were pelleted by centrifugation and suspended in PBS. For cell cycle analysis, each cell sample was suspended in 300 μl PBS/serum-free medium containing 200 μg/ml of saponin (Fluka, NY,USA), 250 μg/ml RNase A (Sigma-Aldrich, MO, USA) and 50 μg/ml propidium iodide, incubated for 30 min at a room temperature and subjected to FACS analysis. At least 10,000 cells were measured per sample. Cell cycle analysis was performed with Coulter EPICS XL Flow Cytometer and WinList and ModFit LT software (Verity Software House). Proliferation was also evaluated by growth curve assay. In the growth medium, 10^5^ cells per 35-mm plate were seeded. Cells were harvested at the indicated time, stained with trypan blue, and counted. Experiments were repeated three times. Propidium iodide (PI) staining was used to determine cell viability. PI is excluded by viable cells but can penetrate cell membranes of dead cells and intercalates into double-stranded nucleic acids. 50 μg/ml propidium iodide (PI) was added to each sample just before analysis and mixed gently. Flow cytometry was performed using the CytoFLEX (Beckman Coulter, CA, USA) and the obtained data were analyzed using CytExpert software version 1.2. To discriminate the live and dead cells, two parameter histogram was used (FL4LOG vs. SLOG). Each sample was analyzed for 50 s and at least 20,000 cells were acquired for analysis. Triplicate counts were obtained for each procedure.

### Statistical analysis

Data are presented as the means with their standard deviations from at least three cell cultures in one experiment. Statistical differences were calculated using the Tukey *t*-test. A level of p < 0.05 was considered to be statistically significant.
